# Has the new Scottish GP contract improved GPs’ working lives in deprived areas? A secondary analysis of two cross-sectional national surveys of GPs’ views in 2018 and 2023

**DOI:** 10.3399/BJGPO.2025.0055

**Published:** 2026-02-11

**Authors:** Lauren Ng, Carey J Lunan, Stewart W Mercer

**Affiliations:** 1 Usher Institute, University of Edinburgh, Edinburgh, UK

**Keywords:** general practice, Scottish GP contract, deprivation, health inequalities, inverse care law, health inequities, primary health care

## Abstract

**Background:**

The new 2018 Scottish GP contract aimed to reduce GP workload and address health inequalities in primary care.

**Aim:**

To compare the working life experiences of GPs working in affluent and deprived areas in 2023, and assess changes since 2018.

**Design & setting:**

Two postal surveys were conducted in 2018 (*n* = 2465, 56% response rate) and 2023 (*n* = 1378, 30% response rate), of all qualified GPs in Scotland.

**Method:**

Secondary analysis of GP working life experiences (job satisfaction, job pressures, negative and positive job attributes) in the most affluent and deprived quintiles. Analysis of covariance (ANCOVA) was used to adjust mean values for GP and practice characteristics that differed significantly between affluent and deprived settings.

**Results:**

In 2023, GPs in affluent areas reported lower job pressures (*P*<0.001) and fewer negative job attributes (*P*<0.001) than GPs in deprived areas in both unadjusted and adjusted analysis. Compared with 2018, GPs in affluent areas in 2023 reported significant improvements in job satisfaction, job pressures, and negative job attributes in unadjusted (*P* = 0.016, *P*<0.001, and *P*<0.001, respectively) and adjusted (*P* = 0.023, *P* = 0.001, and *P*<0.001, respectively) analysis, and positive job attributes in adjusted analysis (*P* = 0.045). In contrast, GPs in deprived areas reported a significant increase in job pressures (*P* = 0.029), with no other changes in working life experiences.

**Conclusion:**

Since the implementation of the 2018 Scottish GP contract, stark contrasts continue to exist in the working life experiences of GPs in affluent areas compared with deprived areas. Targeted strategies are required to address the inverse care law in order to achieve the contract’s intended goals.

## How this fits in

The 2018 Scottish GP contract aimed to reduce health inequalities and GP workload, but evidence of its impact has been limited. While qualitative studies have highlighted challenges in deprived areas, there is limited quantitative evidence to support this. This study compares the working life experiences of GPs in affluent and deprived areas in 2023, and examines changes since 2018, 5 years after the start of the new contract. The findings demonstrate improvements across all domains of working life (job satisfaction, job pressure, negative job attributes, and positive job attributes) for GPs working in affluent areas, but increasing job pressures for GPs in deprived areas (and no changes in the other three domains), emphasising the need for targeted strategies to address the inverse care law in order to achieve the contract’s intended goals.

## Introduction

Scotland has the widest health inequalities and lowest life expectancy in Western Europe, with the gap continuing to widen.^1,2^ These disparities are more pronounced in socioeconomically deprived areas, where the life expectancy is over a decade shorter in the most deprived regions, compared with the more affluent regions, and healthy life expectancy — defined as years lived in good health — is reduced by more than 20 years.^2,3^

Primary care and general practice plays a key role in addressing health inequalities, improving population health, and reducing healthcare costs.^4^ Despite general practice being the largest sector of NHS activity, it receives 6.5% of the total NHS budget in Scotland (compared with approximately 11% 15 years ago),^5^ a figure approximately reflected across the UK.^6–8^ This allocation is particularly significant in deprived areas, where the inverse care law exists and ‘*the availability of good medical care tends to vary inversely with the need for it in the population served*’. ^9^ In such areas of high deprivation, the higher demand for healthcare — driven by factors such as mental health problems and complex multimorbidity, leading to higher consultation rates and poorer health outcomes — places greater pressure on the available resources and workforce supply, which is not matched to the healthcare needs of the population being served.^10,11^

In 2018, the Scottish Government introduced its first GP contract, with stated aims to address health inequalities and alleviate GP workload through the expansion of multidisciplinary teams (MDT).^12^ However, recent qualitative evidence has highlighted ongoing challenges, particularly in deprived areas, in relation to GP workload and health inequalities.^13^ Soon after the implementation of the new Scottish contract, a 2018 national survey demonstrated disparities in working life experiences between GPs in affluent and deprived areas.^14^ No quantitative studies have yet examined how the contract has influenced these experiences in affluent and deprived areas since then.

A follow-up survey was conducted in 2023 using the same working life questions as the 2018 survey, providing an opportunity to assess the impacts of the contract on GP working life experiences 5 years after its implementation.^15^ This study aims to evaluate whether the contract has addressed these disparities by comparing the current working life experiences of GPs in affluent and deprived areas in 2023, and assessing changes from 2018 to 2023.

## Method

### Study design and setting

This study is a secondary analysis of two national cross-sectional surveys conducted in 2018 and 2023, examining the work life experiences of GPs in Scotland.

A postal survey of all qualified GPs in Scotland was conducted in October 2023,^15^ replicating the 2018 survey process.^16^ In 2023, surveys (*n* = 4529) were sent using GPs' names and practice addresses,^17^ with two reminders for non-responders. Unique identifiers enabled follow-up of responders and non-responders. Data collection ended in March 2024.

A total of 2465 out of 4371 (56%) responded to the 2018 survey, and 1378 out of 4529 (30%) to the 2023 survey.^15,16^ The characteristics of responding GPs were very similar in both years, and were broadly nationally representative at both time points.^15,16^

### Instruments used

The 2023 Scottish GP survey employed the same validated measures of working life as the 2018 Scottish GP survey and the English National GP Worklife Survey,^16,18^ which has been described in full elsewhere.^15^ In brief, the survey assessed four key domains of current working life: job satisfaction, job pressures, and positive and negative job attributes.

Deprivation was measured based on the percentage of registered patients classified as ‘very deprived’ by the Scottish Government, defined as those in the top 15% of deprivation as measured by the Scottish Index of Multiple Deprivation (SIMD).^19^ Practices were subsequently categorised into deprivation quintiles, with quintile 1 representing the least deprived areas (affluent) and quintile 5 the most deprived (deprived).

### Data analysis

Survey data from 2018 and 2023 was analysed using SPSS (version 27), with a specific focus on GPs practising in affluent (quintile 1) and deprived (quintile 5) areas. Comparative descriptive analysis was conducted using appropriate parametric or non-parametric tests, depending on the variable type and distribution. Analysis of covariance (ANCOVA) was used for multivariate analysis to assess the variance in responses explained by group differences, applied only when significant differences were observed between groups.

## Results

### GP working life experiences 2023

The characteristics of GPs in affluent and deprived areas from the 2023 survey are shown in Table 1. A significantly higher proportion of GPs in deprived areas were non-White (*P* = 0.019) and more worked seven or more sessions per week (*P*<0.001), compared with those in affluent areas. No other significant differences in demographic or practice-related characteristics were observed.

**Table 1.** Characteristics of GPs working in affluent and deprived areas in 2023

	Affluent (quintile 1) *n* = **369**	Deprived (quintile 5) *n* = **276**	*P*-value
**Age, years: mean (SD)**	47.7 (8.6)	46.6 (0.53)	0.132

**Gender: *n* (%)**	*n* = 366	*n* = 275	0.586
Male	159 (43.4)	125 (45.5)	
Female	206 (56.3)	150 (54.5)	
Other	1 (0.3)	0 (0)	

**Ethnicity: *n* (%)**	*n* = 364	*n* = 273	0.019
White	342 (94.0)	242 (88.6)	
Other	22 (6.0)	31 (11.4)	

**GP position: *n* (%)**	*n* = 368	*n* = 276	0.757
Partnership	310 (84.0)	230 (83.3)	
Non-partner	58 (15.8)	46 (16.7)	

**Sessions per week: *n* (%)**	*n* = 267	*n* = 274	<0.001
<7	219 (82.0)	127 (46.4)	
≥7	148 (55.4)	147 (53.6)	

**Practice list size: *n* (%)**	*n* = 369	*n* = 276	0.443
<5000	134 (36.3)	73 (26.4)	
5000–10 000	152 (41.2)	160 (58.0)	
≥10 000	83 (22.5)	43 (15.6)	

Analysed using Mann–Whitney U, except for age where independent samples *t*-test was applied

In 2023, GPs in affluent areas reported significantly higher job satisfaction (*P* = 0.012), lower job pressures (*P*<0.001), and fewer negative job attributes (*P*<0.001), compared with those in deprived areas (Table 2). After adjusting for ethnicity and number of sessions, differences between the two surveys in job satisfaction were no longer significant. However, job pressures (adjusted *P*<0.001) and negative job attributes (adjusted *P*<0.001) remained significantly lower in affluent areas. Positive job attributes did not differ between the groups in either analysis.

**Table 2.** Differences in GP working life experiences between affluent and deprived areas in 2023

Working life	Affluent unadjusted mean (95% CI)	Deprived unadjusted mean (95% CI)	*P*-value (unadjusted)	Affluent adjusted mean (95% CI)	Deprived adjusted mean (95% CI)	*P*-value (adjusted)
**Job satisfaction**	*n* = 355	*n* = 257	0.012	*n* = 355	*n* = 257	0.102
	5.39 (5.28 to 5.50)	5.23 (5.12 to 5.34)		5.39 (5.29 to 5.49)	5.26 (5.15 to 5.38)	
**Job pressures**	*n* = 347	*n* = 256	<0.001	*n* = 347	*n* = 256	<0.001
	3.36 (3.27 to 3.44)	3.60 (3.51 to 3.70)		3.37 (3.29 to 3.45)	3.61 (3.51 to 3.70)	
**Negative job attributes**	*n* = 358	*n* = 269	<0.001	*n* = 358	*n* = 269	<0.001
	3.74 (3.68 to 3.80)	3.93 (3.86 to 4.00)		3.75 (3.69 to 3.81)	3.91 (3.84 to 3.98)	
**Positive job attributes**	*n* = 357	*n* = 267	0.178	*n* = 357	*n* = 267	0.207
	3.37 (3.32 to 3.43)	3.32 (3.26 to 3.38)		3.37 (3.32 to 3.43)	3.32 (3.26 to 3.38)	

Satisfaction items measured on a 7-point scale (1 = extremely dissatisfied, 7 = extremely satisfied). Work pressure is measured on a 5-point scale (1 = no pressure, 5 = high pressure). Job attributes are measured on a 5-point scale (1 = strongly disagree, 5 = strongly agree). Analysed using independent *t*-test for unadjusted means and *P*-values; and ANCOVA for adjusted means and *P*-values, controlling for ethnicity and number of sessions per week

### Changes in GP working life experiences between 2018 and 2023

Table 3 shows the GP and practice characteristics of those working in affluent and deprived areas for both the 2018 and 2023 surveys. In the affluent-area group, a greater proportion of GPs were working fewer than seven sessions per week in 2023 compared with 2018 (*P*<0.001). No other significant demographic or practice-related differences were found. In the deprived-area group, no significant differences were observed in the GP or practice characteristics between the two surveys.

**Table 3.** Characteristics of GPs in affluent and deprived areas in 2018 and 2023

	Affluent: 2018 survey *n* (%)	Affluent: 2023 survey *n* (%)	*P*-value	Deprived: 2018 survey *n* (%)	Deprived: 2023 survey *n* (%)	*P*-value
**Age, years**	*n* = 454	*n* = 349	0.127	*n* = 459	*n* = 254	0.285
≤40	132 (29.1)	75 (21.5)		125 (27.2)	76 (29.9)	
41–50	150 (33.0)	136 (39.0)		153 (33.3)	88 (34.6)	
>50	172 (37.9)	138 (39.5)		181 (39.4)	90 (35.4)	
**Gender**	*n* = 479	*n* = 366	0.491	*n* = 490	*n* = 275	0.674
Male	196 (40.9)	159 (43.4)		215 (43.9)	125 (45.5)	
Female	283 (59.1)	206 (56.3)		275 (56.1)	150 (54.5)	
Other	0 (0)	1 (0.3)		0 (0)	0 (0)	
**Ethnicity**	*n* = 466	*n* = 364	0.195	*n* = 485	*n* = 273	0.413
White	447 (95.9)	342 (94.0)		439 (90.5)	242 (88.6)	
Other	19 (4.1)	22 (6.0)		46 (9.5)	31 (11.4)	
**GP position**	*n* = 472	*n* = 368	0.274	*n* = 490	*n* = 276	0.468
Partnership	384 (81.4)	310 (84.2)		418 (85.3)	230 (83.3)	
Non-partner	88 (18.6)	58 (15.8)		72 (14.7)	46 (16.7)	
**Sessions per week**	*n* = 475	*n* = 367	<0.001	*n* = 489	*n* = 274	0.599
<7	205 (43.2)	219 (59.7)		217 (44.4)	127 (46.4)	
≥7	270 (56.8)	148 (40.3)		272 (55.6)	147 (53.6)	
**Practice list size**	*n* = 479	*n* = 369	0.291	*n* = 492	*n* = 276	0.294
<5000	201 (42.0)	134 (36.3)		157 (31.9)	73 (26.4)	
5000–10 000	167 (34.9)	152 (41.2)		255 (51.8)	160 (58.0)	
>10 000	111 (23.2)	83 (22.5)		80 (16.3)	43 (15.6)	

Analysed using Mann–Whitney U

In the unadjusted analyses, GPs in affluent areas reported significant improvements in job satisfaction (*P* = 0.016), reduced job pressures (*P*<0.001), and fewer negative job attributes (*P*<0.001) between 2018 and 2023. However, there was no significant change in positive job attributes. After adjusting for the number of sessions, all of these improvements in the affluent group remained significant (adjusted *P*-values: job satisfaction *P* = 0.023, job pressures *P*<0.001, negative job attributes *P*<0.001). Additionally, positive job attributes showed a significant improvement after adjustment (adjusted *P* = 0.045) (Table 4).

**Table 4.** Differences in GP working life experiences between 2018 and 2023 for affluent and deprived areas

Affluent (quintile 1)						
**Working life**	**GP survey 2018 unadjusted mean (95% CI)**	**GP survey 2023 unadjusted mean (95% CI)**	***P*-value (unadjusted)**	**GP survey 2018 adjusted mean (95% CI)**	**GP survey 2023 adjusted mean (95% CI)**	***P*-value (adjusted)**
**Job satisfaction**	*n* = 461	*n* = 360	0.016	*n* = 461	*n* = 360	0.023
	5.22 (5.13 to 5.31)	5.39 (5.28 to 5.50)		5.24 (5.15 to 5.33)	5.40 (5.30 to 5.50)	
**Job pressures**	*n* = 465	*n* = 352	<0.001	*n* = 465	*n* = 352	0.001
	3.55 (3.48 to 3.62)	3.36 (3.27 to 3.44)		3.54 (3.47 to 3.62)	3.36 (3.28 to 3.45)	
**Negative job attributes**	*n* = 475	*n* = 363	<0.001	*n* = 475	*n* = 363	<0.001
	3.89 (3.84 to 3.94)	3.74 (3.68 to 3.80)		3.89 (3.84 to 3.95)	3.75 (3.67 to 3.81)	
**Positive job attributes**	*n* = 475	*n* = 362	0.060	*n* = 475	*n* = 362	0.045
	3.29 (3.23 to 3.35)	3.37 (3.32 to 3.43)		3.30 (3.24 to 3.35)	3.38 (3.32 to 3.44)	
**Deprived (quintile 5)**						
**Working life**	**GP survey 2018 mean (SD)**	**GP survey 2023 mean (SD)**	***P*** **-value**			
**Job satisfaction**	*n* = 485	*n* = 262	0.990			
	5.24 (0.99)	5.24 (0.88)				
**Job pressures**	*n* = 479	*n* = 260	0.029			
	3.49 (0.77)	3.62 (0.74)				
**Negative job attributes**	*n* = 492	*n* = 273	0.366			
	3.86 (0.60)	3.90 (0.55)				
**Positive job attributes**	*n* = 492	*n* = 271	0.595			
	3.29 (0.58)	3.32 (0.51)				

Working life (affluent): analysed using independent *t*-test for unadjusted means and *P*-values; and ANCOVA for adjusted means and *P*-values, controlling for number of sessions per week. Working life (deprived): analysed using independent *t*-test

In contrast, GPs in deprived areas demonstrated an increase in job pressures (*P* = 0.029) in 2023 compared with 2018, but no significant improvements in other working life experiences (Table 4).

## Discussion

### Summary

This study examined GP working life experiences in affluent and deprived areas in 2023 and compared changes between 2018 and 2023. In 2023, GPs working in affluent areas reported higher job satisfaction, lower job pressures, and fewer negative job attributes compared with those working in deprived areas. After adjusting for GP characteristics that differed significantly between the high and low deprivation groups (ethnicity and the number of sessions worked), job pressures and negative job attributes remained lower in affluent areas, but differences in job satisfaction were no longer significant. Positive job attributes did not differ between the two groups in either unadjusted or adjusted analyses.

Between 2018 and 2023, GPs working in affluent areas saw improvements across most working life domains in both unadjusted and adjusted analyses, including job satisfaction, job pressures, and negative job attributes. While positive job attributes showed no change in the unadjusted analysis, a significant improvement was observed after adjusting for the number of sessions worked (*P* = 0.045). In contrast, GPs in deprived areas reported an increase in job pressures, with no significant improvements in other working life domains.

### Strengths and limitations

This study utilised nationally representative data from two cross-sectional surveys in 2018 and 2023, facilitating direct comparison of GP working life experiences soon after and 2 years following the 2018 Scottish GP contract’s implementation. The use of validated measures to assess GP working life experiences also provides methodological consistency, and comparison with other studies.

Limitations include a lower response rate in 2023 (30%) compared with 2018 (56%), which may introduce non-response bias. However, the demographic characteristics of responders were similar across both years.^15^ Previous research suggests that non-responding GPs are more likely to face greater time constraints and job pressures, potentially underestimating the extent of these challenges.^20^ Reasons for this lower response rate are unclear but may relate to increased workload in general practice and the fact that the survey was somewhat longer in 2023 than 2018 owing to additional questions relating to multidisciplinary teams.

### Comparison with existing literature

These findings reinforce the ongoing presence of the inverse care law in Scotland, where the higher levels of multimorbidity and social complexity in deprived areas place greater demands on primary care,^21^ and are associated with poorer access, shorter GP consultation times, higher GP stress, and lower patient enablement than affluent areas.^10,11,21–24^ Workforce shortages and underfunding relative to need further challenge these issues.^10,11^

These patterns have also been observed across the UK and internationally.^24–27^ In Denmark, GPs in deprived areas report higher rates of burnout,^25^ while in Australia, market-driven healthcare forces exacerbate inequalities, limiting private providers’ ability to meet the needs of patients in deprived areas.^26^

The impact of the COVID-19 pandemic on working life also warrants consideration. The pandemic had notable influence on general practice, including worsening time pressures and lower staff morale.^13,28^ Lower socioeconomic groups were disproportionately affected by the health and economic effects of the pandemic,^29^ which may have also amplified GP pressures in deprived areas. Nevertheless, while intensified by the pandemic, these challenges stem from pre-existing inequalities in chronic disease burden and social determinants of health.^30^

### Implications for research and practice

Despite the 2018 Scottish GP contract’s aims to reduce GP workload and address health inequalities,^12^ our findings suggest it has not yet achieved these goals for GPs working in deprived areas.^12,13^ Indeed, our results would suggest that (in terms of the working life domains we have measured) the contract has ‘worked’ for GPs working in more affluent areas but ‘not worked’ for GP working in more deprived areas. The long-term effects of the pandemic may have contributed to these disparities, which were apparent in 2018 but clearly persist in 2023.^29,30^

At the time of writing, phase two negotiations of the Scottish GP contract are underway, which will refine funding models and resource allocation for general practice.^12^ This presents an opportunity to ensure funding models better reflect the complexity and intensity of workload and support equitable resource distribution, particularly in the areas of greatest need.

Various interventions to address the inverse care law in Scotland have been undertaken, including enhancing financial support, targeting specific health conditions, and improving the ability to provide high-quality generalist care.^31^ For example, the CARE Plus intervention, which focused on longer consultations and continuity of care for patients with multimorbidity in deprived areas, was both cost-effective (£12 224 per quality-adjusted life year gained) and improved quality of life.^32^ However, a significant gap between policy ambitions and their sustainable delivery remains.^31^ Research has also shown that sustained NHS investment in more socioeconomically deprived areas can reduce mortality gaps and improve outcomes, reinforcing the need for long-term, needs-based funding.^33^

In addition, a clearer understanding of ‘unmet need’ in general practice could improve funding allocations.^34^ Recent research has used routine national data to quantify unmet need by comparing GP contact time between patients from affluent and deprived groups, demonstrating the potential of data-driven approaches to inform equitable funding models.^35^ Further research is needed to develop measurable proxies of unmet need to better inform resource allocation.^36^

In conclusion, this study highlights the persisting disparity in working life experiences between GPs in affluent and deprived areas, despite the introduction of the 2018 Scottish GP contract. While GPs working in affluent areas report improvements, job pressures in deprived areas have increased, with no significant changes in other working life experiences. These findings underscore the need for further policy changes to address the inverse care law and fulfil the contract’s aim to reduce health inequalities in Scotland.

## Data

The dataset relied on in this article is available from the corresponding author on reasonable request.
